# Are There Limits in Explainability of Prognostic Biomarkers? Scrutinizing Biological Utility of Established Signatures

**DOI:** 10.3390/cancers13205087

**Published:** 2021-10-12

**Authors:** Frank Emmert-Streib, Kalifa Manjang, Matthias Dehmer, Olli Yli-Harja, Anssi Auvinen

**Affiliations:** 1 Predictive Society and Data Analytics Lab, Faculty of Information Technology and Communication Sciences, Tampere University, 33720 Tampere, Finland; kalifa.manjang@tuni.fi; 2 Department of Computer Science, Swiss Distance University of Applied Sciences, 3900 Brig, Switzerland; matthias.dehmer@umit.at; 3 Department of Mechatronics and Biomedical Computer Science, UMIT, 6060 Hall in Tyrol, Austria; 4 College of Artificial Intelligence, Nankai University, Tianjin 300350, China; 5 Computational Systems Biology, Faculty of Medicine and Health Technology, Tampere University, 33720 Tampere, Finland; olli.yli-harja@tuni.fi; 6 Institute for Systems Biology, Seattle, WA 98195, USA; 7 Institute of Biosciences and Medical Technology, 33720 Tampere, Finland; 8 Unit of Health Sciences, Faculty of Social Sciences, Tampere University, 33720 Tampere, Finland; anssi.auvinen@tuni.fi

**Keywords:** prognostic biomarker, causal model, computational biology, biostatistics, genomics, survival analysis

## Abstract

Prognostic biomarkers can have an important role in the clinical practice because they allow stratification of patients in terms of predicting the outcome of a disorder. Obstacles for developing such markers include lack of robustness when using different data sets and limited concordance among similar signatures. In this paper, we highlight a new problem that relates to the biological meaning of already established prognostic gene expression signatures. Specifically, it is commonly assumed that prognostic markers provide sensible biological information and molecular explanations about the underlying disorder. However, recent studies on prognostic biomarkers investigating 80 established signatures of breast and prostate cancer demonstrated that this is not the case. We will show that this surprising result is related to the distinction between causal models and predictive models and the obfuscating usage of these models in the biomedical literature. Furthermore, we suggest a falsification procedure for studies aiming to establish a prognostic signature to safeguard against false expectations with respect to biological utility.

## 1. Introduction

According to [1] a prognostic biomarker is defined as follows:

“A prognostic biomarker is one that indicates an increased (or decreased) likelihood of a future clinical event, disease recurrence or progression in an identified population.”

This definition is rather broad and does not specify the nature of a biomarker which can either refer to the measurement of protein levels [2], gene expressions [3], gene mutations [4] or other types of biological information. Furthermore, a biomarker can be a single entity, e.g., the mutation in one particular gene, or it can refer to a set of such entities in which case one speaks of biomarkers or a set thereof. Importantly, if one has a set of biomarkers, usually, the entities in such a set are of a similar type, i.e., they all represent protein levels or gene expressions or genetic mutations. Although theoretically possible, practically, mixed sets of biomarkers with multiple types are rare.

**Figure 1:**
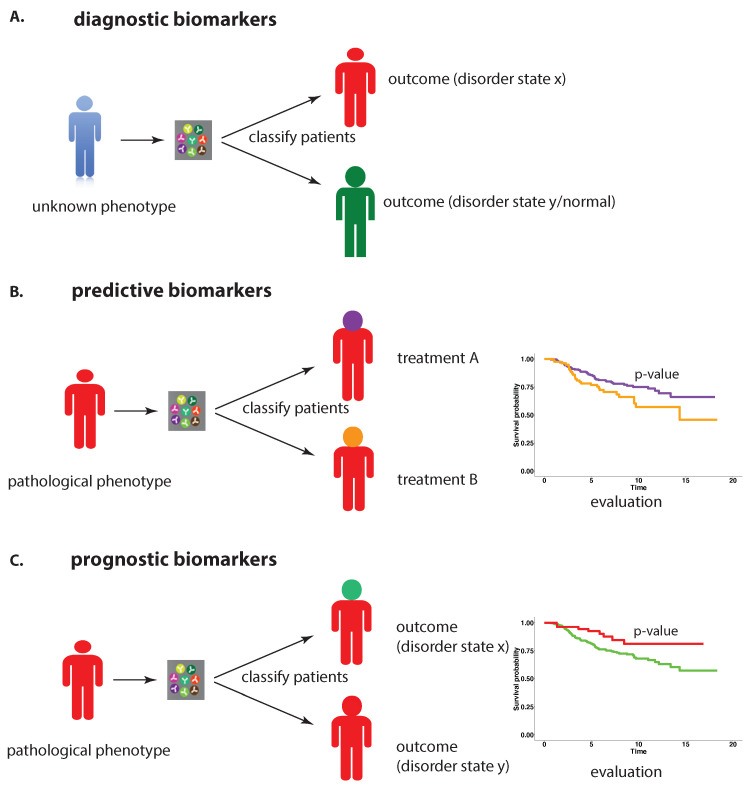
An idealized overview of three types of biomarkers and their application purpose. (**A**) diagnostic biomarkers, (**B**) predictive biomarkers, (**C**) prognostic biomarkers.

Regardless of the specific type of biomarker, the above definition implies that biomarkers can be used to categorize patients, obtaining this way an ‘*identified population*’, and that each patient group represents a defined outcome or progression of the disorder. Simplifying our discussion to a two binary classification of patients with detectable differences in the progression of the disorder in these two groups; see Figure 1C. Usually, such groups may exhibit a different *(survival) time to event*, where event’ and ‘survival time’ are context-specific. More precisely, an ‘event’ could refer to death, end of progression-free survival or end of disease-free survival from either the onset of a tumor, time of infection or date of a surgery. Each end-point can be used to define a time duration of survival for individual patients. Depending on the nature of the ‘event’ these survival times are called overall survival (O-death), disease-specific survival (death from the disorder), progression-free survival (PFS-worsening of disorder), relapse-free survival (RFS-recurrence of disorder) and disease-free survival (DFS-onset of disorder). As a consequence, statistical methods have been developed for identifying differences in the survival times between patient groups, which is practically accomplished by comparing Kaplan Meier curves via statistical hypothesis tests of the corresponding two patient groups [5].

Therefore, in order to study or identify prognostic biomarkers, one needs: (i) at least two distinct groups of patients, where (ii) each group represents a particular prognostic phenotype of a disorder. Examples from the literature studied, e.g., stage II and stage III colon cancer [6], grade 2 to 4 glioma patients [7] or triple-negative vs non-triple negative breast cancers [8]. It is interesting to note that prognostic biomarkers are also used when no specific prognostic phenotype (either via staging or grading) of disorders are defined but only its heterogeneity, see, e.g., ref. [9] for chronic lymphocytic leukaemia or [10] for human ‘immunodeficiency virus type 1’ (HIV-1). In these cases, patient groups with different survival are used even if no a priori definition of prognostic phenotype is available.

Importantly, the definition of the prognostic phenotype should not be based on treatment (e.g., chemotherapy or medication) because this would correspond to predictive biomarkers [1] nor on the presence of the disorder because this would correspond to diagnostic biomarkers. Instead, the groups need to correspond to contrasting outcomes of the disorder. In Figure 1, we visualize the different application purposes for the three different types of biomarkers. In the following, we will focus on prognostic biomarkers.

## 2. Discovery Procedure Underlying Prognostic Biomarkers

From a Pubmed search, one finds that there are over 80,000 articles that investigate general prognostic biomarkers. Despite this enormous number of articles, all of these studies have a common underlying design which can be summarized by a general discovery procedure [11,12]. Briefly, this discovery procedure can be described by the following five steps:

1. Generation of gene expression data
2. Preprocessing of the data
3. Selection of biomarkers
4. Categorization of patient samples
5. Assessment of the biomarkers

**Figure 2:**
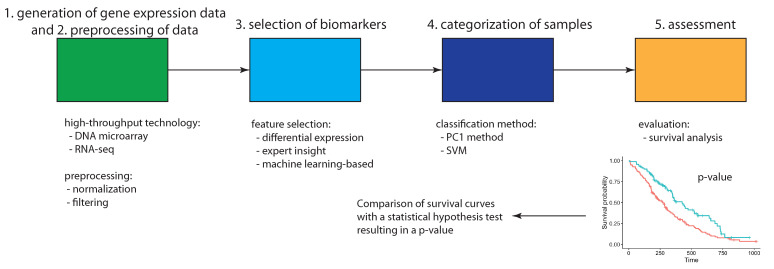
General procedure used by studies for establishing prognostic biomarkers. The first three steps involve the generation and preprocessing of expression data and the selection of potential signatures. The fourth step uses the signature for a categorization of patient samples followed by an evaluation of the quality of this categorization by means of survival analysis.

The procedure shown in Figure 2 corresponds to the generally applied approach whenever prognostic biomarkers are established or studied. The former means the demonstration that a set of biological features has the predictive capability of distinguishing patient groups with a different prognostic phenotype [13]. Essentially, all studies follow these steps differing mainly in methodological aspects. For instance, different approaches are used for selecting signature genes or other biological features as potential biomarkers. Most of these implement context-specific biological propositions, which are portrayed as important for the disorder under investigation. One frequently used approach involves the identification of differentially expressed genes or hub genes in regulatory networks, e.g., [14,15,16,17].

Another example relates to the categorization of the patients (samples from patients) utilizing a variety of different classification methods. For instance, in [18] the PC1 method is used based on principal component analysis whereas in [19] a support vector machine (SVM) is utilized. Interestingly, for assessing the prognostic value of signature genes, only one approach is used, namely a survival analysis [20]. Specifically, the comparison of different Kaplan Meier survival curves is conducted by using a statistical hypothesis test that detects significant differences in these curves. Usually, only two survival curves are compared, corresponding to two patient groups with contrasting prognostic phenotype, however, extensions to more groups are possible. Among the most frequently used tests is the Mantel-Haenszel test (aka log-rank test) [21].

In the following, we focus mainly on gene expression signatures of breast and prostate cancer, but our general point may extend to other cancer types and disorders.

## 3. Problems in the Interpretation of Prognostic Biomarkers

Prognostic biomarkers are utilized in two complementary ways. In the following, we call these the *predictive utility* and *biological utility* of biomarkers.

1. Predictive utility: The predictive utility of prognostic biomarkers means that biomarkers are used to categorize patients according to their prognostic phenotype (see Figure 1C).
2. Biological utility: The biological utility of prognostic biomarkers means that biomarkers are used to provide biological insights into disease development and progression.

We would like to emphasize that usually this distinction is not made explicitly but implicitly [22]. Examples for such a dual usage are abundant in the literature and some specific instances thereof are provided by markers that even entered the clinical practice [23], e.g., MammaPrint [24], Oncotype DX [25] or Prosigna/Pam50 [26].

Both usages appear very natural, at first, because how could biomarkers with a predictive utility not be useful for a biological explanation of a disorder? However, in the statistics community, one distinguished between two types of models. One type is called an explanatory model or causal model, whereas the other one is called predictive model [27,28]. Importantly, an explanatory model is more informative than a predictive model because even though both models can make predictions, only an explanatory model provides a sensible explanation for the functioning of the underlying system about which predictions are made. A prime example for an explanatory model is a causal Bayesian network, in contrast, deep neural networks are examples for prediction models. For this reason, the latter type of models are sometimes called *black-box* models [29].

Recent studies of prognostic biomarkers have revealed that such a distinction is also of relevance for biomarkers. Specifically, in [18] the authors studied 48 prognostic signatures of breast cancer, derived in separate, dedicated studies, and showed that when performing a random selection of genes one can always find sets of genes that have the same predictive abilities as the original signatures. Similar results have been earlier observed in [30] where genes were ranked according to their correlation with survival outcome and successive (non-overlapping) groups of genes have been used for classifying patients. Despite a decaying probability for finding such groups of correlated genes for more distant groups, the authors demonstrated the existence of such groups even for genes that do not rank at the top. The study by [30] is related to [31] where the influence of varying training sets has been investigated resulting in varying gene sets that show the closest correlation with survival. Importantly, the difference between [18,30,31] is that the former study used a random selection of genes whereas the latter performed a correlation-based selection. However, regardless of the selection mechanism, all studies found sets of genes with predictive performance similar to the original selection.

**Figure 3:**
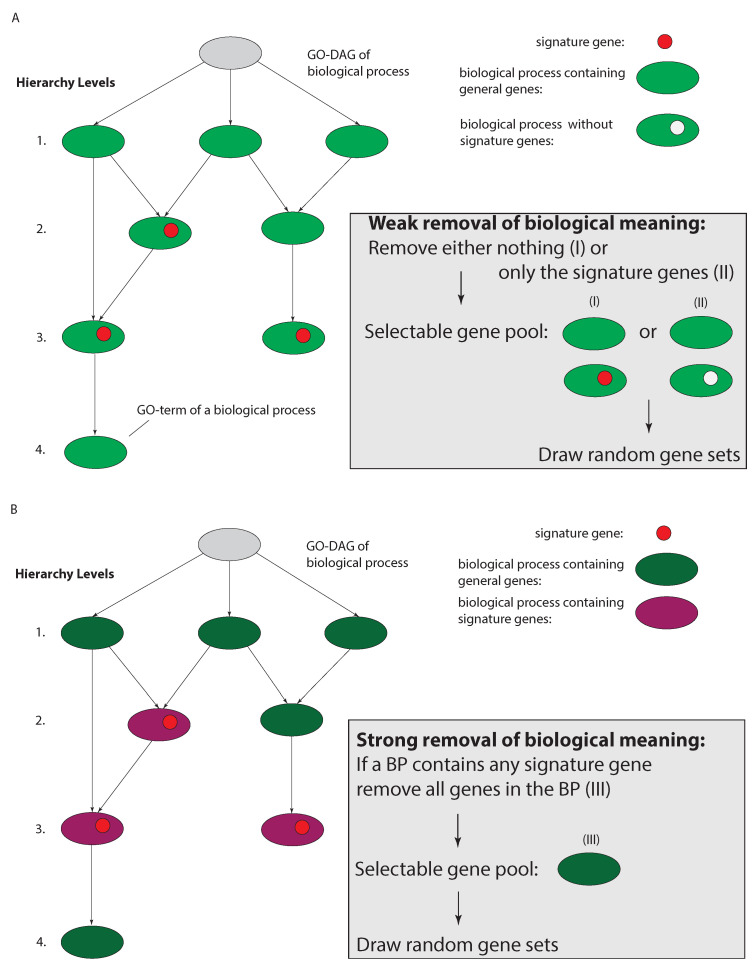
Visualization of three different ways to remove ’biological information’ from a pool of available genes. The hierarchical trees on the left-hand side are showing directed acyclic graphs (DAG) corresponding to the entire GO database of biological processes (BPs). For a weak removal of biological meaning there are two ways, one allows genes from all BPs (I) and one that removes only the signature genes (II) (**A**). In contrast, a strong removal selects only genes from GO-terms that do not contain any signature gene (**B**).

Extending to disorders with complex etiology rather than Mendelian heredity such as cancer, in [22,32] the biological meaning of biomarkers has been further challenged. Specifically, instead of only selecting genes randomly to form new signature sets, as in [18], in [22,32] all signature genes and all genes involved in the same biological processes as the signature genes were removed (Remark: Even genes involved in proliferation were removed). In Figure 3, we show a visualization of the conceptual difference of these studies. Figure 3A shows a simplified GO-DAG, which is a hierarchically organized directed acyclic graph (DAG), containing all GO-terms of biological processes (BP) of an organism where each GO-term contains a certain number of genes. Also, genes in a signature (shown in red) belong to a number of GO-terms representing biological processes. Figure 3B shows the biological processes containing such signature genes highlighted in magenta. While the study by [18] performed a random sampling from all genes, which can include genes in the original signature (corresponding to the weak removal I), the studies in [22,32] removed not only all signature genes but in addition all genes belonging to the same biological processes as the signature genes (corresponding to the strong removal III). Hence, the available pool of selectable genes is much smaller and those genes do not have any GO-terms in common with the signature genes.

Interestingly, despite the further reduction in the size of the pool of selectable genes, the studies in [22,32] showed that even among those remaining genes there exist random gene sets that perform similarly in the prognostic prediction task. Such gene sets are called surrogate gene sets. Importantly, due to the removal of all genes that share biological processes with the original signature genes, all remaining genes do not share any biological meaning with such a signature. Since this holds also for any random gene set drawn from these remaining genes this demonstrates that such random gene sets have an entirely different biological meaning compared to the signature genes. Due to these differences, the procedure in [18] performs a weak removal (by diluting selectable genes) of biological meaning whereas the procedure in [22,32] perform a strong removal of biological meaning.

All of these studies demonstrate that the dual usage of prognostic biomarkers, i.e., for a predictive utility and biological utility, is not justified. Especially the studies in [22,32] eliminated systematically the possibility that random gene sets could *accidentally* carry the same (or similar) biological meaning as the original signatures by excluding all such genes from the available pool of selectable genes. Returning to the statistical distinction of models discussed above, as a consequence of these studies, none of the procedures used for identifying prognostic biomarkers could establish causal models. Instead, they all lead to predictive models which do not allow to draw conclusions about the underlying biology or disease etiology.

For reasons of clarity, we would like to remark that despite the fact that no one (explicitly) assumes cancer is a Mendelian disease, nevertheless, cancer is usually studied this way. In contrast, in [22,32] cancer is studied as a non-Mendelian disease by explicitly exploiting the hierarchical network structure connecting the genes as provided by gene ontology [33]. This simple, yet efficient mechanism enables a new way for the falsification of biological utility.

## 4. Falsification Procedure to Test Biological Meaning

In order to safeguard against false statements about the biological utility of prognostic biomarkers, we suggest the following falsification procedure [32]. This procedure corresponds to a formalization of the strong removal procedure discussed above (see Figure 3C), and provides a Gene Removal Procedure (GRP).

1. *G*: total number of genes in a cancer dataset.
2. Remove proliferation genes, PG, from *G*. The set PG contains proliferation genes. This gives a new set of genes $G^{*}$ with $G^{*}$ = G∖PG.
3. $BM:\{g_{1},...,g_{m}\}$. BM is the gene signature and $g_{1},...,g_{m}$ are the genes in the corresponding signature.
4. Map the genes in BM to GO-terms. This gives:
  (1) $$\begin{array}{c} BM=\left\{g_{1},...,g_{m}\right\}\to \left\{GO_{1},...,GO_{t}\right\}. \end{array}$$
  Note, each gene can be connected to more than one GO-term. For this reason m≤t.
5. Map the GO-terms to genes. This gives:
  (2) $$\begin{array}{c} GO_{i}\to g\left(i\right)=\left\{g_{1}\left(i\right),...,g_{k}\left(i\right)\right\}. \end{array}$$
  for all GO-terms *i* with i∈{1,⋯,t}.
6. Delete all the genes in $D=\cup_{i\in \{1,\cdots ,t\}}g\left(i\right)$ from *G*. This results in a new gene set given by $G^{\prime }$= $G^{*}\setminus D$.
7. From $G^{\prime }$, sample new sets of random genes of size |BM| and perform the prognostic task. The resulting gene sets are called *random gene sets* (RGS). This is repeated *B* times.
8. Apply a Bonferroni correction (which is the most conservative correction) to the *p*-values and assess the performance for a significance level of α.

For easy usage of the above falsification procedure, we implemented an R package called KARL available from Github that gives the functionality described above.

In case the falsification procedure does not result in any surrogate gene set with the same prognostic prediction capabilities, the BM signature might have a biological meaning that deserves to be discussed. We would like to highlight that the procedure above does only safeguard against lightly made statements but does not directly prove biological utility. Hence, the cautious formulation regarding a potential biological meaning.

### dividual genes in this signature give?

In our above discussion, we used the expression ’biological meaning’ of signatures without giving a precise definition. In the following, we fill this gap by providing a formal definition [22].

The foundation for our definition of ’biological meaning’ is given by gene ontology (GO) [33]. Specifically, GO provides GO-terms for genes, e.g., in the category biological process (BP). That means for each gene, $g_{i}$, one can assign a list of GO-terms in the form

(3) $$\begin{array}{c} g_{i}\to \left\{GO_{1}\left(i\right),\cdots ,GO_{t}\left(i\right)\right\}. \end{array}$$

In the following, all GO-terms are from the category BP. We define the biological meaning, ‘*M*’, of one gene as the list of these GO-terms, e.g.,

(4) $$\begin{array}{ccc} \mathrm{biological}\;\mathrm{meaning}\;\mathrm{of}\;\mathrm{gene}\;g_{i}:M\left(g_{i}\right) & := & \{GO_{1}\left(i\right),\cdots GO_{t}\left(i\right)\}. \end{array}$$

For a given biomarker signature consisting of *m* genes, i.e., $BM=\{g_{1},...,g_{m}\}$, we define the meaning of the entire signature as the union of all GO-terms of the individual genes in this signature given by

(5) $$\begin{array}{c} \mathrm{biological}\;\mathrm{meaning}\;\mathrm{of}\;\mathrm{gene}\;\mathrm{set}\;BM:M\left(BM\right)=M\left(\left\{g_{1},\cdots ,g_{m}\right\}\right):=\cup_{i=1}^{m}M\left(g_{i}\right) \end{array}$$

That means M(BM) represents the biological meaning of a set of genes. For our analysis the biological meaning of an individual set is of less importance, instead, we are interested in the comparison of two sets, i.e., we are interested in the biological meaning of

(6) $$\begin{array}{c} \mathrm{biological}\;\mathrm{meaning}\;\mathrm{common}\;\mathrm{in}\;\mathrm{set}\;S_{1}\;and\;S_{2}:M\left(S_{1},S_{2}\right):=M\left(S_{1}\right)\cap M\left(S_{2}\right) \end{array}$$

for two gene sets $S_{1}$ and $S_{2}$. It is clear that for two arbitrary gene sets $S_{1}$ and $S_{2}$ the intersection can assume subsets of the underlying GO-terms which could lead to a partial overlap. However, we are not studying arbitrary gene sets. Instead, one gene set is given by the signature itself, i.e., BM, whereas the second gene set is constructed by our Gene Removal Procedure (GRP) defined above (for technical details see [32]) constructing random gene sets (RGS) with the property

(7) $$\begin{array}{c} M(RGS)\cap M(BM)=\emptyset . \end{array}$$

Hence, all random gene sets we study result in zero common GO-terms with the signature genes. For this reason, we are safe to say that the overlap in biological meaning of a signature and the genes in a RGS is zero-based on the information provided by the gene ontology [33].

## 5. Discussion

After reaching conceptual clarity about the dual utility of prognostic biomarkers, we discuss now some implications thereof that could be helpful for future studies.

*I. Any study claiming to have found prognostic biomarkers with a causal interpretation should demonstrate this by application of the falsification procedure [32]:* Applying the falsification procedure provides an extra level of stringency that will facilitate distinguishing if prognostic biomarkers constitute a predictive model or a causal model. This complements general reporting guidelines, e.g., given in [34,35]. It should be highlighted that also predictive models could be of great utility for the clinical practice, however, by explicating the lack of a causal meaning considerable confusion and the study of potentially misleading directions can be avoided. Given the fact that the general purpose of biomarkers is the usage in hospitals or clinics, any reduction in confusing aspects should be desirable.

*II. The presentation of prognostic biomarkers should focus on the demonstrated ability of the model:* Depending on the outcome of the analysis suggested in I., the presentation of prognostic biomarkers should only focus on the demonstrated ability of the model. An immediate implication of this is not to present an extensive discussion of the biological meaning of the genes contained in such a set of prognostic biomarkers when the causal connection between genes and outcome has not been rigorously demonstrated. Such information is not only unhelpful but even counterproductive in potentially misguiding future experiments [36]. Instead, the emphasis of a discussion should be on the predictive utility of signatures when biological utility cannot be established.

*III. Use of biological information is no guarantee of biological meaningfulness:* Probably everyone is aware that an ‘association’ does not establish a ‘causation’ between two entities. A prominent example thereof is the correlation coefficient, which fails to distinguish these effects. However, in the context of biomarkers, correlations are frequently used for arguing that there is a connection between gene expression and survival outcome, see, e.g., [30,31]. As a consequence of this or similar arguments all current biomarkers we are aware of are derived in this way (see also Section ‘Discovery procedure underlying prognostic biomarkers’). Unfortunately, this does not correspond to a causal analysis [37,38] in the strict sense but merely to associations. Hence, even formally, current procedures do not even aim at introducing approaches that establish a causal connection between biomarkers and survival outcomes but provide only information about associations.

The above discussion provides an independent argument from a different angle to understand why established prognostic biomarkers of breast and prostate cancer do not carry explanatory information about the underlying disorder [22,32]. What is unclear at this point is if it is possible to design causal models that would establish prognostic gene expression biomarkers with such an explanatory power or if this is, for some reason, not feasible for breast and prostate cancer.

*IV. The falsification procedure does not address other problems known for prognostic biomarkers:* We would like to emphasize that known problems of biomarkers like the lack of robustness or a small overlap among similar signatures are not addressed by the falsification procedure [31]. Instead, the falsification procedure aims at reducing the risk of making false statements about the biological meaning of a biomarker set.

## 6. Conclusions

Prognostic biomarkers are considered important for the clinical practice because of their potential to allow stratification of patients by prognosis for optimising the choice of treatment. However, we are still facing fundamental problems in their general understanding. In this paper, we raise awareness of a new problem that relates to the biological meaning of established prognostic biomarkers based on recent findings for breast and prostate cancer. We found that discovery procedures currently used for deriving prognostic biomarkers do not establish causal models and for this reason do not provide explanatory information about the underlying disease biology. This has been demonstrated for 80 established signatures [22,32].

In order to reduce confusion about the biological meaning of signatures and to prevent potential misuse, e.g., for guiding follow-up studies, we suggest a falsification procedure that can be applied to any study aiming to establish prognostic signatures. This falsification procedure can provide an additional level of stringency that could complement existing protocols for general prognostic markers [34,35]. If the biological meaning of signatures for other cancer types and disorders other than breast and prostate cancer, and if markers other than gene expression suffer from such limitations remains to be seen. However, our falsification procedure can also be applied to such settings for obtaining clarity.

## Data Availability

The data presented in this study are available on request from the corresponding author.
